# Oral health-related beliefs among a sample of pregnant women in Southwestern Ontario: a descriptive study

**DOI:** 10.3389/froh.2024.1485815

**Published:** 2024-10-23

**Authors:** Yasaman Mohammadi Kamalabadi, M. Karen Campbell, Robert Gratton, Alexia Athanasakos, Myriam Haddad, Abbas Jessani

**Affiliations:** ^1^ Department of Epidemiology and Biostatistics, Schulich School of Medicine and Dentistry, Western University, London, ON, Canada; ^2^ Department of Pediatrics, Schulich School of Medicine & Dentistry, Western University, London, ON, Canada; ^3^ Department of Obstetrics/Gynecology, Schulich School of Medicine & Dentistry, Western University, London, ON, Canada; ^4^ Children’s Health Research Institute, Lawson Health Research Institute, London, ON, Canada; ^5^ Lawson Health Research Institute, London, ON, Canada; ^6^ Department of Dentistry, Schulich School of Medicine and Dentistry, Western University, London, ON, Canada

**Keywords:** oral health, pregnant women, Ontario, attitude to health, dental care, healthcare utilization

## Abstract

**Introduction:**

Unfavorable beliefs about oral health and dental treatment during pregnancy can lead to the avoidance of dental care and the underutilization of dental services, adversely affecting adherence to good oral hygiene practices and, consequently, the health of the fetus. This study investigated the commonly held oral health beliefs among pregnant women in Southwestern Ontario, Canada.

**Methods:**

Participants were recruited from the Family Medicine Obstetrics Clinic in London, Ontario, Canada. Eligible participants were pregnant women aged 18 or older, excluding those unwilling to participate. Participants completed a 33-item self-administered questionnaire, including three open-ended questions about oral health beliefs and their impacts on pregnancy, which were analyzed for this study. Thematic analysis in NVivo identified key patterns, while analysis determined the most common beliefs and the degree of diversity in responses. Responses were categorized into sub-themes, and the frequency and percentage of each category were calculated.

**Results:**

A total of 130 participants met the inclusion criteria. Among them, 40.7% (*n* = 46) believed that oral health impacts their child's health, 48.2% (*n* = 53) believed that dental treatment affects fetal health, and 64.4% (*n* = 76) believed that pregnancy influences their oral health. Many beliefs regarding oral health during pregnancy regarded infection risks. Participants held negative beliefs about the effects of antibiotics, analgesics, and dental x-rays during pregnancy. Common beliefs about the impacts of pregnancy on oral health included developing conditions, such as tooth sensitivity, caries, and gingivitis, as well as a loss of minerals to the fetus.

**Conclusion:**

This study revealed important misconceptions and concerns about oral health and dental treatment among pregnant women in Southwestern Ontario. The findings highlighted the need for enhanced oral health education for pregnant women to address these misconceptions and promote proper care during pregnancy. Healthcare providers are encouraged to focus on dispelling myths, emphasizing the safety of necessary dental treatments, and reinforcing the significance of maintaining good oral health for maternal and fetal well-being.

## 1 Introduction

Pregnant women are at an increased risk of developing oral conditions including periodontal disease and gingivitis due to hormonal changes and the increased pH in the oral cavity during pregnancy (1). Oral diseases may exacerbate systemic inflammation and comprise the ability to maintain adequate nutrition and overall health—factors that are essential for both maternal and fetal well-being (2). Furthermore, oral diseases during this period adversely impact important aspects of quality of life, including psychological, physical, and functional aspects (3). Maintaining adequate oral health must be advocated not only to benefit the well-being of the expecting mother but also to support to general health and development of the fetus (4).

Despite the literature emphasizing the importance of maintaining good oral health during pregnancy, access to and utilization of dental care in this period remains suboptimal worldwide (5). The various barriers that pregnant women face, including logistical challenges, financial constraints, and minimal awareness of dental care importance during pregnancy, contribute to the difficulty in accessing dental care, with unfavorable beliefs about oral health and the safety of dental treatment appearing to be the most frequent and significant barriers (6, 7). In a recent systematic review, many misbeliefs were identified regarding the safety of certain dental procedures, including the use of local anesthetics, medical prescription, and restorative procedures, with concerns over dental radiographs being the most prevalent among the included studies (8). Another prevalent misconception was regarding the safety of the mother and fetus during regular dental check-ups, where women often opted for the avoidance of preventative dental due to the belief of its potential harm to the fetus (8). The review further reported periodontal diseases and dental caries as a “norm” that was expected by women during pregnancy (8).

Such strong unfavorable beliefs can be shaped by societal attitudes, family influences, inadequate education, and, in some environments, the advice of healthcare providers—directly contradicting the guidelines developed by the American and Canadian Dental Associations emphasizing preventative dental care during pregnancy (9–12). Culture, elder family members, peers from social networks, and low economic and educational statuses are the most common sources of such misbeliefs during pregnancy (13).

Unfavorable beliefs about oral health and dental treatment during pregnancy can be directly associated with the avoidance of dental treatments and the underutilization of dental services (8, 14). Such beliefs can profoundly impact the importance of adequate oral health maintenance, and, in turn, the adherence to good oral hygiene practices, which can be directly associated with the health of the fetus (13). Understanding these unfavorable perceptions about oral health is crucial for developing and implementing risk-based preventative intervention and raising oral health awareness during pregnancy to dispel adverse information. To the authors’ knowledge, research on oral health-related beliefs of pregnant women is limited, particularly in the context of Southwestern Ontario. While minimal studies have specifically addressed this issue in Canada, it is important to recognize the country's multicultural diversity, which may influence oral health beliefs and behaviors. Previous research (15, 16) highlights the need for region-specific studies. This descriptive study aims to address this gap by investigating the oral health beliefs of pregnant women in Southwestern Ontario.

## 2 Materials and methods

This study is part of a cross-sectional study that investigates the unmet oral health needs and patterns of dental care utilization by pregnant women in Southwestern Ontario. This study was approved by the Western University Health Science Research Ethics Board (Review Reference: 2022-121440-70801) and the Lawson Health Research Institute (Approval number: R-22-505).

### 2.1 Participant recruitment and data collection

Participants were recruited from the Family Medicine Obstetrics Clinic at the London Health Sciences Centre in London, Ontario, Canada. This clinic focuses on providing antenatal care to pregnant women who lack a family physician or other antenatal care provider. Eligible participants were pregnant women aged 18 or older, with the only exclusion criterion being unwillingness to participate. Before the study commencement, a 30-minute meeting was held with clinic nurses and the clinic assistant to discuss the study objectives, consent form, questionnaire, and recruitment process. Given the descriptive and preliminary nature of the study, we collected samples from two cohorts of pregnant women from November 8 to December 6, 2022, and from May 1 to May 30, 2023, with a response rate of 86.7%. This methodology was also preferred to ensure the feasibility of the staff and site in the process of data collection. The gap in recruitment periods resulted from the limited availability of new patients at each clinic session. As the study advanced, most pregnant women attending the clinic were returning for follow-up visits, reducing the pool of new eligible participants. Consequently, a pause in recruitment was implemented to allow for the actual of additional new patients. Pregnant women were approached by the clinic assistant to complete a 33-item self-administered questionnaire. The questionnaire was provided in English, Arabic, and Spanish. For participants who could not read or speak in these listed languages, an interpreter provided by the London Health Sciences Centre, as part of routine care, assisted with completing the questionnaire. The development of the questionnaire as well as the quantitative data from 30 of the 33 items in the questionnaire have been detailed elsewhere (17). For this illustrative study (18), data from the three open-ended questions were used to get a general perspective of commonly held oral health beliefs during pregnancy (Q31-Q33) (17). These qualitative questions were selected to address all potential beliefs identified in an initial literature review, which was later published as a systematic review of unfavorable beliefs (8).

### 2.2 Data analysis

The quantitative data including demographic information and responses to the three open-ended questions categorized as “Yes,” “No,” or “Not Sure,” were analyzed first by one researcher (YMK) using descriptive statistics to identify initial trends and patterns. This preliminary quantitative analysis was conducted independently before any thematic analysis of the qualitative data to prevent potential bias from influencing the qualitative findings.

For the qualitative analysis of the open-ended questions, thematic analysis was conducted independently by two researchers (MH and YMK). Positive responses were read multiple times by the two reviewers (MH and YMK) to aid in the familiarization of the content. Preliminary codes were assigned to significant text segments that captured key ideas and patterns related to the three main themes. These codes were then grouped into broader sub-themes representing the primary ideas expressed by respondents. Member checking was performed by one reviewer (AJ) to ensure the accuracy and reliability of the generated sub-themes. Additionally, content analysis was performed to identify the most common beliefs and the degree of consensus or diversity in the responses. Responses were categorized based on the sub-themes identified during thematic analysis, and the frequency and percentage of each category were calculated.

## 3 Results

Overall, 150 women were approached to participate in the study and 130 (86.7%) responded to the survey while responding to the belief questions pertinent to access to oral health.

Table 1 provides the general characteristics of a sample of pregnant women recruited from the Family Medicine Obstetrics Clinic in Southwestern Ontario. The majority of participants were between 22 and 34 years old (*n* = 96; 73.6%) and married (*n* = 86; 66.2%). Almost half were in the third trimester of their pregnancy (*n* = 60; 46.2%). A higher proportion of participants completed college or university education (*n* = 84; 64.6%) and had an income of more than $80,000 (*n* = 28; 22.1%). The majority were born in a country other than Canada (*n* = 88; 67.7%), lived in Canada for less than five years (*n* = 58; 70.3%), and did not classify themselves as a refugee (*n* = 73; 83.0%).

**Table 1:** General characteristics of a sample of pregnant women recruited from the family medicine obstetrics clinic, Southwestern Ontario (17).

Characteristics		Distribution of responses *n* (%)
Age (*n* = 130)	18–21	7 (5.4)
	22–34	96 (73.6)
	35–43	27 (20.8)
Trimester (*n* = 130)	First	20 (15.4)
	Second	50 (38.5)
	Third	60 (46.2)
Marital status (*n* = 130)	Married	86 (66.2)
	Living common law	31 (23.9)
	Other	13 (10.0)
Education (*n* = 130)	Did not complete high school	8 (6.2)
	High school diploma or equivalency certificate	22 (16.9)
	Some community college/university, trade school/vocational college	16 (12.3)
	Complete college or university	84 (64.6)
Birth country (*n* = 130)	Canada	42 (32.3)
	Other	88 (67.7)
Years of living in Canada (*n* = 82)	=<5 years	58 (70.3)
	>5 years	24 (29.3)
Refugee (*n* = 88)	No	73 (83.0)
	Yes	15 (17.1)
Income (*n* = 127)	<$20.000	23 (18.1)
	$20.000-$40.000	27 (21.3)
	$40.000-$60.000	20 (15.8)
	$60.000-$80.000	12 (9.5)
	>$80.000	28 (22.1)
	Prefer not to answer	17 (13.4)

Table 2 shows the frequency of responses to questions regarding oral health during pregnancy. Less than half reported that oral health can affect the health of the fetus (*n* = 46; 40.7%). More than half reported that dental treatment can impact the oral health of the fetus (*n* = 53; 48.2%) and pregnancy can affect their own oral health (*n* = 76; 64.4%).

**Table 2:** Frequency distributions of oral health-related beliefs among a sample of pregnant women recruited from a family medicine obstetrics clinic in Southwestern Ontario.

Concepts	Responses	Distribution of responses *n* (%)
Oral health and health of the fetus (*n* = 113)	Yes	46 (40.7)
	No	54 (47.8)
	Not sure	13 (11.5)
Dental treatment and health of the fetus (*n* = 110)	Yes	53 (48.2)
	No	45 (40.9)
	Not sure	12 (10.9)
Oral health and pregnancy (*n* = 118)	Yes	76 (64.4)
	No	27 (22.9)
	Not sure	15 (12.7)

Table 3 provides the most common themes and sub-themes pertinent to oral health beliefs, along with illustrated examples from the participants. Beliefs regarding the impact of oral health during pregnancy generated four sub-themes: (1) risk of infection, (2) adverse pregnancy outcome, (3) general health of the mother and 4) other. The respondents expressed their beliefs of *possible infections* and *adverse pregnancy outcomes* such as *pre-mature childbirth* due to pregnancy.

**Table 3:** Main themes and sub-themes of beliefs about oral health during pregnancy among a sample of pregnant women recruited from the Family Medicine Obstetrics Clinic, Southwestern Ontario.

Concept	Sub-themes	*n*	Verbatim examples
Beliefs about the impact of oral health on pregnancy (*n* = 46)	Risk of infection	16	“Bacterial infection might be absorbed by the fetus”
			“Possible serious infection”
			“If not treated it could affect their mouth and cause worse effects if not treated”
	Adverse pregnancy outcomes	5	“Gingivitis and tartar can cause premature childbirth”
	General health of the mother	5	“Maintaining a good oral health may reduce the chance of diseases and also mental wellbeing”
			“Poor dental care can lead to infections carried through body via blood”
	Other	9	“Baby's oral health comes from mother”
			“Bad teeth can lead to issues with eating and pretty much everything else”
			“If I feel pain, my child will feel it”
			“By the bacteria that I can transmit by the saliva”
Beliefs about the impact of dental treatment on pregnancy (*n* = 53)	Negative impacts of Medications	12	“If I take antibiotic, it can be dangerous for my child”
			“Chemicals might be harmful for the fetus”
	Negative impacts of Dental X-rays	11	“X-ray can be harmful”
	Negative impacts of Anesthetics	10	“I think anesthesia can be harmful”
			“I believe it can as they cannot use freezing or anesthesia on me due to pregnancy”
			“Anesthesia can affect the baby”
			“X-ray or anesthetic agents are not good in pregnancy”
	Positive aspects of dental treatment	7	“Eradicating an infection would probably be beneficial”
			“Without proper care it can make things more difficult”
			“…it can help quality of life”.
			“In a positive way by catching something before it's too late”
			“Helping issues get resolved or not get worse”
	Treatment-specific risk	5	“Depending on treatment, certain things are not recommended during pregnancy”
			Only if I need surgery or anesthesia
	Other	5	“Treatment can affect pregnancy if poor hygiene occurs in dental clinic”
			“Having dental treatment can increase complications during pregnancy which can affects the fetus with tooth extraction, high chance of bleeding may happen”
			“Irritability due to pain”
			“High chance of bleeding may happen”
			“Maybe temporary bacteremia after a cleaning”
Beliefs about the impact of pregnancy on oral and dental health (*n* = 76)	Gingivitis and gum bleeding	27	“I know gingivitis is a concern”
			“Swollen gums, etc. due to pregnancy”
			“I know it could produce bleeding gums”
	Dental caries, tooth sensitivity and pain	19	“Pregnancy increases the cavities”
			“Weakens teeth enamel and affects gum health”
			“I may be more prone to cavities or lose teeth due to lack of adequate calcium”
	Loss of minerals by the fetus	16	“Loss of calcium and potassium”
			“I feel more sensitive, and we lose calcium due to absorption by the baby”
			“Decalcification or some other nutrients”
			“Because the baby is needing and taking everything from mom”
			“Because of calcium level getting low”
			“Having less calcium can degenerate your teeth maybe”
			“I may be more prone to cavities or lose teeth due to lack of adequate calcium”
			“Weakening of teeth/bone because of calcium deficiency”
	Morning sickness and gag reflux	9	“Stomach acid from morning sickness can affect your oral/dental health”
	Hormonal changes	7	“Hormones affect oral health”
			“…and hormones can also affect dental health”
			“Due to cavities + gums swelling because of hormonal changes”
	Diet	4	“Eating habits change”
	Delaying dental treatments during pregnancy	2	“Pregnancy can postpone certain dental procedures and cause them to possible become worse before pregnancy is over and you can receive treatment”
			“Along with immunity, dental health is compromised during pregnancy”

Beliefs regarding the impact of dental treatment on pregnancy generated six sub-themes: (1) use of medications, (2) use of dental x-rays, (3) use of anesthetics, (4) positive aspects of dental treatment, (5) treatment-specific risk, and (6) other. Most commonly, the respondents held beliefs regarding the *harmful effects of antibiotics, analgesics, and x-rays*, while others attributed potential risks to *specific procedures* such as *surgery* during pregnancy. Regarding the positive aspects of dental treatments during pregnancy, respondents indicated that *eradicating [dental] infections, catching something [dental problems] before it's too late, helping [dental] issues get resolved or not get worse,* and *eradicating [dental] infection* would be beneficial for them and could improve their *quality of life*.

Beliefs regarding the impact of pregnancy on oral and dental health generated seven sub-themes: (1) gingivitis and gum bleeding; (2) dental caries, tooth sensitivity, and pain; (3) loss of minerals by the fetus, (4) morning sickness and gas reflux; (5) hormonal changes; (6) diet; and (7) delaying dental during pregnancy. Developing oral conditions such as *tooth sensitivity, dental caries, bleeding gums, and gingivitis due to pregnancy* were widely reported by the participants. Another important sub-theme identified was the loss of calcium by the fetus where the respondents commonly believed to be *more prone to cavities or lose teeth due to lack of adequate calcium, lose calcium due to absorption by the baby, having less calcium can degenerate your teeth maybe,* and other similar beliefs. Participants believed that *morning sickness* and *stomach acid* were associated with oral conditions during pregnancy, while *hormonal and dietary changes* were also the reasons for changes in their oral health. *Delaying dental during pregnancy* was also reported by participants.

## 4 Discussion

The results of this study identified the commonly held oral health beliefs among pregnant women recruited from the Family Medicine Obstetrics Clinic in Southwestern Ontario. The majority of pregnant women perceived themselves to have good, very good, or excellent oral health and recognized oral health as very or extremely important.

When beliefs regarding oral health during pregnancy were assessed, several main themes were identified. Beliefs regarding the impact of oral health during pregnancy generated four sub-themes, with the most common being risk of infection and adverse pregnancy outcomes. Similar results were identified by Liu et al. (19) and Murphey (9) as many of their participants feared dental clinics and dental treatment due to the perception that the air and procedures would lead to fetal bacterial infections. The reason for this could be the impact of oral health conditions on overall well-being and quality of life during the prenatal period (8). Periodontal disease can lead to localized inflammation of the supportive periodontal structures, and untreated dental decay can lead to odontogenic infections. While evidence remains inconclusive, some studies have suggested that these oral conditions may be associated with adverse pregnancy outcomes, such as preterm birth and low birth weight (20). Additionally, oral health conditions can exacerbate discomfort and stress during pregnancy, further affecting maternal health and fetal development (21). However, these potential issues can be prevented by maintaining adequate oral health through behavioral modifications (i.e., preventative dental screening, limiting sugar consumption, and a healthy diet) and promptly addressing dental issues (8, 22). According to the American and Canadian Dental Association guidelines, visiting an oral health provider during pregnancy is not only safe but also essential for maintaining adequate oral health (11). Delaying treatments requiring prompt attention such as periodontal care, extractions, root canals, or restorations may result in more complex problems (11, 12). To prevent tooth decay, oral infections, and tooth loss, semi-annual dental examinations and cleaning, as well as daily brushing and flossing are recommended, especially during pregnancy (11, 12).

Beliefs regarding the impact of dental treatment on pregnancy generated six sub-themes, including negative beliefs regarding the use of medication, dental x-rays, and anesthetics; positive aspects of dental treatment; and negative beliefs regarding treatment-specific risk, with the former being the most common. This is consistent with findings reported in a systematic review by Kamalabadi and colleagues (8), which found that participants in several studies reported to believe that medications pertinent to dental treatment should be avoided because of harm to the mother or fetus. When necessary, antibiotics such as penicillin, erythromycin, and cephalosporins have been reported safe to use during pregnancy; however, tetracycline, vancomycin, and streptomycin are contraindicated (23). Tetracycline causes permanent discoloration of teeth in the fetus and affects the calcification of the bones and teeth, while tetracycline and vancomycin cause nephrotoxicity and ototoxicity, respectively (23, 24). Therefore, medications, of any kind, should be administered following consultation with healthcare providers to ensure safety and the mitigation of any adverse effects on the oral health and well-being of the mother and fetus.

The findings indicated that the second most common sub-theme regarding beliefs about the impact of dental treatment on pregnancy was negative perceptions of dental x-rays. Kamalabadi et al. reported in their systematic review that the prevalence of unfavorable beliefs about adverse pregnancy outcomes due to dental x-ray exposure ranged from 31% to 93%, with many participants suggesting that radiography could lead to miscarriage (8). The safety of dental radiography during pregnancy has been a controversial topic for decades, but recent literature has reported that, if performed properly, the amount of ionizing radiation produced is so minimal that it is unlikely to reach the teratogenic threshold, and thus, unlikely to cause *in utero* birth defects (25). Although dental x-rays emit minimal levels of radiation, there is still a potential risk as the effect of radiation exposure during pregnancy is dependent on the gestational age of the fetus, with greatest resistance during the second and third trimesters and more susceptible in the first (26). While some argue that the benefits of diagnosing and preventing dental issues outweigh the risks (25), others advocate for postponing non-essential x-rays until after pregnancy or taking extra precautions to minimize exposure (27). Such a perspective of treating to avoid adverse dental outcomes was also highlighted by this study as participants considered oral health and dental treatment important during pregnancy.

Beliefs regarding the impact of pregnancy on oral and dental health generated seven sub-themes, with the most common being gingivitis and gum bleeding; dental caries, tooth sensitivity, and pain; and loss of minerals by the fetus. According to the quantitative findings of this sample, a significant number of participants reported having dental conditions such as periodontal disease and dental caries during pregnancy (17). Similar findings have been reported by Islam & Haque (28), where the most common oral problems emphasized during pregnancy were gingivitis, periodontitis, tooth erosion, xerostomia, tooth decay, and pregnant tumours. As such, having dental conditions is often considered ‘normal’ during pregnancy and pregnant women are expected to cope with these oral health challenges (29). An additional “norm” reported was the loss of minerals due to the fetus ‘taking up’ nutrients during pregnancy. However, this is not supported by the literature (30). Nawabi and colleagues (31) reported that the degree of knowledge of pregnant women is associated with levels of education, culture, socioeconomic status, and other psychosocial factors. Since our study was conducted in a prenatal clinic with varied social determinants of health, we hypothesize that this could be positively associated with unfavorable beliefs about loss of minerals to the fetus; however, a larger study design is recommended to confirm such a hypothesis.

Our results highlighted several perceptions and beliefs regarding oral healthcare during pregnancy. Some of these views, particularly pertaining to the loss of calcium or the normalization of dental conditions due to pregnancy, are not supported by the evidence. On the contrary, our participants exhibited positive aspects of oral healthcare during pregnancy, such as seeking dental treatment promptly and understanding hormonal changes and their implications on oral health, among others. The results of the study underscore the importance of integrating oral health as part of primary healthcare for pregnant women. A study by Israel (32) shared that the integration of midwives and community health workers in clinical settings may significantly improve prenatal health, reduce maternal morbidities, and increase healthy birth and breastfeeding outcomes. Such integration should be conducted with interdisciplinary education and collaboration among undergraduate health learners, such as dental students, aiding the translation to interprofessional collaboration in the real world.

### 4.1 Limitations

The limitations of our analysis include potential bias in the qualitative analysis, as one researcher (YMK) was involved in both the quantitative analysis and assisted with the qualitative coding which could have influenced the interpretation of the qualitative data. However, we tried to mitigate this bias by involving a second independent reviewer (MH) and member checking (AJ) to ensure the accuracy and reliability of the qualitative analysis. Additionally, the process of simultaneous data collection and separate analysis can be complex, especially if the findings from the two components diverge, and may oversimplify nuanced beliefs when categorizing qualitative responses. Additionally, a small percentage of respondents did not have English as their first language. Although Spanish and Arabic translations were provided, there remains the potential for interpretative bias in our results. Another limitation of this study is the lack of generalizability due to the small sample size from an inner-city clinic. We recommend that future studies involve larger and more diverse samples to enhance the applicability of findings. Additionally, future research should consider assessing predictors of oral health beliefs and practices, including socioeconomic factors, cultural influences, and access to healthcare services, to provide a more comprehensive understanding of these issues across different populations. Despite these limitations, our study highlights some of the commonly held beliefs among pregnant women, specifically in the North American and Canadian context.

## 5 Conclusion

This study highlights the prevalent misconceptions and concerns regarding oral health and dental treatment among pregnant women in Southwestern Ontario. Despite recognizing the importance of oral health, many participants held beliefs that could deter them from seeking necessary dental care during pregnancy, potentially impacting both maternal and fetal well-being. The findings underscore the need for targeted oral health education and better integration of dental care into prenatal services. Addressing these misconceptions through evidence-based guidance can help ensure that pregnant women receive the essential oral healthcare needed for a healthy pregnancy.

## Data Availability

The raw data supporting the conclusions of this article will be made available by the authors, without undue reservation.
